# Spatiotemporal Analysis of Influenza in China, 2005–2018

**DOI:** 10.1038/s41598-019-56104-8

**Published:** 2019-12-23

**Authors:** Yewu Zhang, Xiaofeng Wang, Yanfei Li, Jiaqi Ma

**Affiliations:** Center for Public Health Surveillance and Information Service, Chinese Center for Disease Control and Prevention, Beijing, China

**Keywords:** Influenza virus, Risk factors

## Abstract

Influenza is a major cause of morbidity and mortality worldwide, as well as in China. Knowledge of the spatial and temporal characteristics of influenza is important in evaluating and developing disease control programs. This study aims to describe an accurate spatiotemporal pattern of influenza at the prefecture level and explore the risk factors associated with influenza incidence risk in mainland China from 2005 to 2018. The incidence data of influenza were obtained from the Chinese Notifiable Infectious Disease Reporting System (CNIDRS). The Besag York Mollié (BYM) model was extended to include temporal and space-time interaction terms. The parameters for this extended Bayesian spatiotemporal model were estimated through integrated nested Laplace approximations (INLA) using the package R-INLA in R. A total of 702,226 influenza cases were reported in mainland China in CNIDRS from 2005–2018. The yearly reported incidence rate of influenza increased 15.6 times over the study period, from 3.51 in 2005 to 55.09 in 2008 per 100,000 populations. The temporal term in the spatiotemporal model showed that much of the increase occurred during the last 3 years of the study period. The risk factor analysis showed that the decreased number of influenza vaccines for sale, the new update of the influenza surveillance protocol, the increase in the rate of influenza A (H1N1)pdm09 among all processed specimens from influenza-like illness (ILI) patients, and the increase in the latitude and longitude of geographic location were associated with an increase in the influenza incidence risk. After the adjusting for fixed covariate effects and time random effects, the map of the spatial structured term shows that high-risk areas clustered in the central part of China and the lowest-risk areas in the east and west. Large space-time variations in influenza have been found since 2009. In conclusion, an increasing trend of influenza was observed from 2005 to 2018. The insufficient flu vaccine supplements, the newly emerging influenza A (H1N1)pdm09 and expansion of influenza surveillance efforts might be the major causes of the dramatic changes in outbreak and spatio-temporal epidemic patterns. Clusters of prefectures with high relative risks of influenza were identified in the central part of China. Future research with more risk factors at both national and local levels is necessary to explain the changing spatiotemporal patterns of influenza in China.

## Introduction

Influenza is associated with notable mortality and morbidity worldwide, as well as in China^1–3^. The behaviours of major epidemics and pandemics of influenza were complicated due to dramatic genetic changes, subtype circulation, wave patterning and virus replacement^4^.

Influenza vaccination is the most effective means to prevent infection, severe disease and mortality^5^. The World Health Assembly recommends vaccinating 75% of key risk groups against influenza^6^. Although seasonal influenza vaccination was introduced in 1998, influenza vaccination is not yet included on the National Immunization Program (NIP) in China^7^. The average national vaccination coverage was reported to be just 1.5–2.2% between 2004 and 2014^7,8^. The overall number of flu vaccines approved for sale by China’s National Institute for Food and Drug Control (NIFDC) has decreased in recent years^9,10^. The low coverage rate and reduction in flu vaccine supplementation have raised much concern about the increased risk of influenza incidence in China.

Although new emerging influenza virus types and subtypes, such as avian influenza A H5N1^11–14^, influenza A (H1N1)pdm09^15–17^, and influenza A H7N9^18,19^, have been reported continuously in China, the disease burden of influenza has been dominated by A(H3N2), A(H1N1)pdm2009 influenza viruses, pre-pandemic A(H1N1) or influenza B in recent years, which account for the majority of cases^20^. The influenza A(H1N1)pdm2009 virus was first introduced to mainland China on May 9, 2009^21^, and has been one of the dominant viruses in the seasonal influenza epidemics since then^20^. The effect of newly emerging influenza A(H1N1)pdm2009 viruses on the geographic patterns and temporal trends of influenza across the whole country is still unknown.

Spatial and spatiotemporal disease mapping are widespread approaches in the data analysis of disease surveillance data. The most popular model of spatial disease mapping was proposed by Besag *et al*.^22^ and developed further by several other researchers^23,24^. By adding terms for a linear^25–27^ or nonparametric trend in time and time-space interactions^25,28,29^, the baseline model was extended to use in the spatiotemporal case^25^.

The influenza surveillance system is a major data source for monitoring and evaluating the transmission and evolution of influenza^30–32^. Two major surveillance systems, the Sentinel Influenza-Like Illness (ILI) Surveillance System and the Chinese Notifiable Infectious Diseases Reporting System (CNIDRS), have been widely used for influenza surveillance and research in China^16,30,33,34^.

In this study, we aimed to describe an accurate spatiotemporal pattern of influenza at the prefecture level in mainland China from 2005 to 2018 and explore the risk factors using Bayesian spatiotemporal disease mapping tools and data from CNIDRS.

## Results

### Descriptive analysis

**Figure 1:**
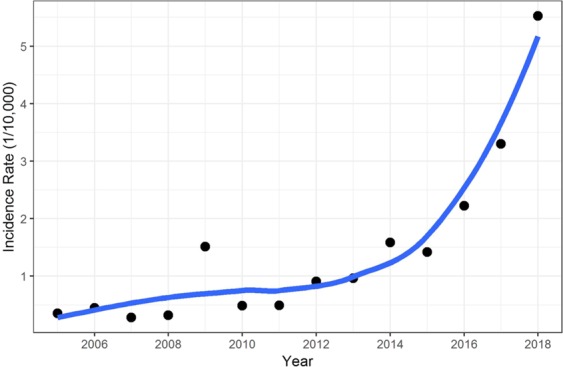
The incidence rate of influenza per 10,000 population with an overlying loess smoothing line from 2005–2018.

Of the 2,702,226 influenza cases that were reported in mainland China via CNIDRS between January 1st, 2005, and December 31st, 2018, 55.8% were male and 44.2% were female. The proportions of cases in age groups 0 to 9 years, 10 to 19 years, 20 to 59 years and 60+ years were 46.9%, 14.8%, 20.8%, and 8.8%, respectively. The yearly incidence rates were 0.35, 0.44, 0.28, 0.32, 1.49, 0.48, 0.49, 0.91, 0.96, 1.59, 1.44, 2.24, 3.31, and 5.51 per 10,000 population from 2005 to 2018 (Fig. 1).

**Figure 2:**
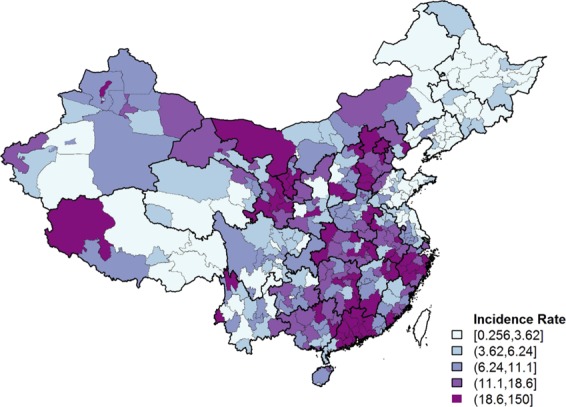
The incidence rate of influenza of prefectures from 2005 to 2018 in China.

The overall incidence rates of influenza from 2005 to 2018 by prefecture are displayed in Fig. 2.

### Model selection for spatiotemporal models

Table 1 presents the DIC components for the four models. Model 4 offered the best trade-off between model fit and complexity. For this reason, we focus on the results from Models 4 and 5 in the following analysis.

**Table 1:** Deviance information criterion (DIC) for five spatiotemporal models.

Model	$\bar{D}$	*pD*	DIC
Model 1*	1129876.4	363.9	1130240.2
Model 2**	1129876.6	363.9	1130240.5
Model 3†	1129876.5	363.9	1130240.4
Model 4‡	35202.0	4467.3	39669.2
Model 5#	34664.3	4522.7	39187.0

Abbreviations: D, posterior mean of the deviance; pD, the number of effective parameters; DIC, the deviance information criterion, as a measure of the trade-off between model fit and complexity.

Note: Model terms used in four models include an intercept (α); a spatially unstructured random effect term (*ν*_*i*_); a spatially structured conditional autoregression term (*υ*_*i*_); uncorrelated time (*γ*_*j*_); a first-order random walk-correlated time variable (*γ*_1*j*_); and an interaction term for time and place (*δ*_1*j*_). *θ*_*ij*_ represents the relative risk of area i at time j.

^*^Model 1, convolution + uncorrelated time (time IID), e.g., $\log ({\theta }_{ij})=\alpha +{\nu }_{i}+{\upsilon }_{i}+{\gamma }_{1j}$, where.

^**^Model 2, convolution + 1st order random walk correlated time (time RW1), e.g., $\log ({\theta }_{ij})=\alpha +{\nu }_{i}+{\upsilon }_{i}+{\gamma }_{1j}$.

^†^Model 3, convolution + 1st order random walk correlated time (time RW1) + uncorrelated time (time IID), e.g., $\log ({\theta }_{ij})=\alpha +{\nu }_{i}+{\upsilon }_{i}+{\gamma }_{1j}+{\gamma }_{j}$.

^‡^Model 4, convolution + 1st order random walk correlated time (time RW1) + space-time interaction term with uncorrelated prior for the interaction term, e.g., $\log ({\theta }_{ij})=\alpha +{\nu }_{i}+{\upsilon }_{i}+{\gamma }_{1j}+{\delta }_{ij}$.

^#^Model 5, model 4 + covariates, e.g., $\log ({\theta }_{ij})={\rm{\alpha }}+{\sum }_{k=1}^{n}{\beta }_{k}{x}_{k}+{\nu }_{i}+{\upsilon }_{i}+{\gamma }_{1j}+{\delta }_{ij}$.

### Covariates associated with the reported incidence cases of influenza

The fixed effects of covariates in the univariate Poisson models, multivariate adjusted Poisson model, and multivariate adjusted spatiotemporal model are shown in Table 2. The crude odds ratios (ORs) and adjusted ORs in both the univariate Poisson models and multivariate adjusted Poisson model are statistically significant. After adjusting for other covariates, a spatially unstructured random effect term (*v*_*i*_), a spatially structured conditional autoregression term (*υ*_*i*_), a first-order random walk-correlated time variable (*γ*_1*j*_), and an interaction term for time and place (*δ*_i*j*_) in the multivariate adjusted spatiotemporal model, the flu vaccines (per million doses), flu surveillance protocols, rate of influenza A (H1N1)pdm09, latitude and longitude still remain statistically significant. Holding all other covariates to zero and adjusting for spatiotemporal variation, every one million increase in the number of influenza vaccines for sale approved by the China Food and Drug Administration was associated with a 12.7% decrease in the influenza incidence risk (95% CI = 0.825–0.923). Similarly, the new update of the influenza surveillance protocol in 2017 was related to a 65.6% increase in the influenza incidence risk (95% CI = 1.097–2.496) compared to the protocol used in 2005 to 2008. For every 10% increase in the rate of influenza A (H1N1)pdm09 among all processed specimens from ILI patients, there was a 19.5% increase in the influenza incidence risk (95% CI = 1.005–1.413). Every one degree increase in the latitude and longitude was associated with a 1.5% (95% CI = 0.980~0.991) and 0.2% (95% CI = 0.997~0.999) increase in the influenza incidence risk, respectively.

**Table 2:** Risk analysis of covariates associated with reported cases of influenza.

Covariates	Crude OR (95% CI)^*^	Adjusted OR (95% CI)^**^	Adjusted OR (95% CI)^†^
Flu vaccines (per million doses)^‡^	0.528(0.527~0.529)	0.645(0.644~0.647)	0.873(0.825~0.923)
Flu surveillance protocols^#^			
Version 1 (2005–2008)	1 [Reference]	1 [Reference]	1 [Reference]
Version 2 (2009–2016)	3.366(3.349~3.383)	4.614(4.588~4.640)	1.045(0.819~1.331)
Version 3 (2017–2018)	11.79(11.73~11.85)	8.381(8.332~8.431)	1.656(1.097~2.496)
Rate of influenza A (H1N1)pdm09^¶^	1.149(1.148~1.151)	1.117(1.114~1.120)	1.195(1.005~1.413)
Percentage of influenza A (H1N1)pdm09^††^	1.206(1.205~1.207)	0.969(0.968~0.970)	1.015(0.958~1.076)
Population density (/km2)	18.29(18.13~18.46)	5.597(5.546~5.649)	2.475(0.642~9.543)
Latitude (degree)	0.940(0.940~0.940)	0.953(0.952~0.953)	0.985(0.980~0.991)
Longitude (degree)	0.998(0.997~0.998)	0.998(0.998~0.998)	0.998(0.997~0.999)

Abbreviations: OR, odds ratio; CI, confidence interval.

^*^Univariate Poisson analysis models.

^**^Multivariate adjusted Poisson analysis model, which included all variables in the univariate analysis models.

^†^Multivariate adjusted spatiotemporal models, which included all variables in the univariate analysis models; an intercept (α); a spatially unstructured random effect term (*ν*_*i*_); a spatially structured conditional autoregression term (*υ*_*i*_); a first-order random walk-correlated time variable (*γ*_1*j*_); and an interaction term for time and place (*δ*_*ij*_).

^‡^Total number of flu vaccines approved for sale by China’s National Institute for Food and Drug Control (NIFDC), which were rescaled to one million doses as one unit. Data were collected from NIFDC.

^#^The influenza surveillance protocols used included three versions: Version 1 for 2005 to 2008, Version 2 for 2009 to 2016, and Version 3 for 2017 to 2018.

^¶^The rate of influenza A (H1N1)pdm09 was calculated by dividing the number of specimens of positive influenza A (H1N1)pdm09 viruses by the number of specimens processed from the influenza likely illness (ILI) cases. The rate was rescaled to 10% changes as one unit. Data were collected from FluNet (www.who.int/flunet), Global Influenza Surveillance and Response System (GISRS).

^†††^The percentage of influenza A (H1N1)pdm09 was calculated by dividing the number of specimens of positive influenza A (H1N1)pdm09 viruses by the total number of specimens of influenza-positive viruses. One unit change equals a 10% change in influenza A (H1N1)pdm09.

### The spatial and temporal effects in spatiotemporal models with covariates

#### The spatial effects

**Figure 3:**
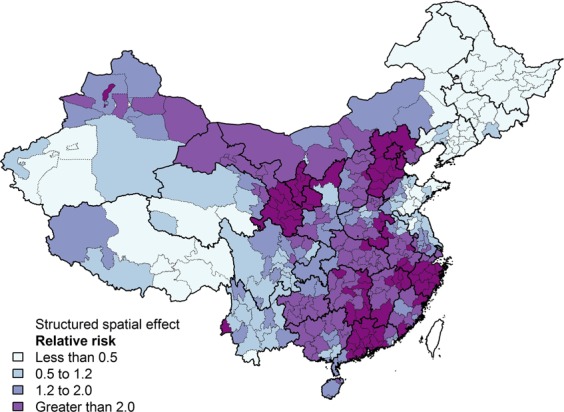
Map of the spatially structured relative risk (${e}^{{\upsilon }_{i}}$), spatiotemporal model of influenza incidence risk with covariates, China Prefectures, 2005–2018. Note: The linear terms in the model of spatiotemporal model of influenza incidence risk with covariates were $\log ({\theta }_{ij})={\rm{\alpha }}+{\sum }_{k=1}^{n}{\beta }_{k}{x}_{k}+{\nu }_{i}+{\upsilon }_{i}+{\gamma }_{1j}+{\delta }_{ij}$, which included all variables in the univariate analysis models; an intercept (α); a spatially unstructured random effect term (ν_i_); a spatially structured conditional autoregression term (υ_i_); a first-order random walk-correlated time variable (γ_1j_); and an interaction term for time and place (δ_ij_).

**Figure 4:**
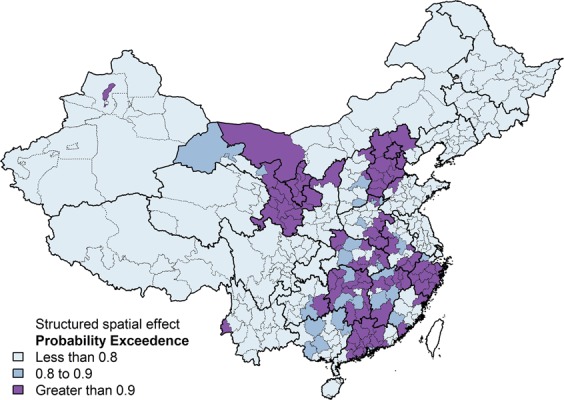
Map of the posterior probabilities of spatially structured relative risk ($${e}^{{\upsilon }_{i}}$$) > 1.0, spatiotemporal model of influenza incidence risk with covariates, China Prefectures, 2005–2018. Note: The linear terms in the model of spatiotemporal model of influenza incidence risk with covariates were $$\log ({\theta }_{ij})={\rm{\alpha }}+{\sum }_{k=1}^{n}{\beta }_{k}{x}_{k}+$$$${\nu }_{i}+{\upsilon }_{i}+{\gamma }_{1j}+{\delta }_{ij}$$, which included all variables in the univariate analysis models; an intercept (α); a spatially unstructured random effect term (ν_i_); a spatially structured conditional autoregression term (υ_i_); a first-order random walk-correlated time variable (γ_1j_); and an interaction term for time and place (δ_ij_).

The map of the spatially structured conditional autoregression term demonstrated areas of spatial patterning and similarity among prefectures. The spatially structured relative risk and posterior probabilities of spatially structured relative risk greater than 1.0 are presented in Figs. 3 and 4, respectively.

**Figure 5:**
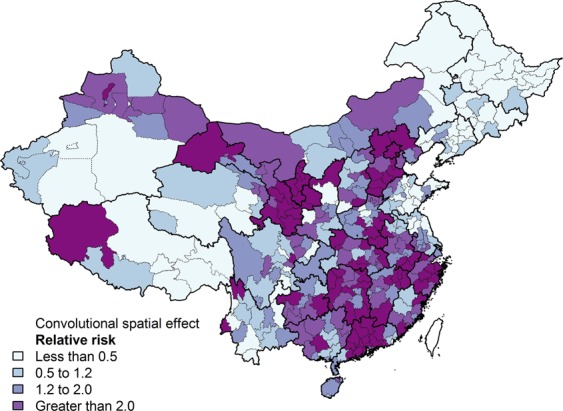
Map of the convolutional spatial relative risk (${e}^{{\upsilon }_{i}+{\nu }_{i}}$), spatiotemporal model of influenza incidence risk with covariates, China Prefectures, 2005–2018. Note: The linear terms in the model of spatiotemporal model of influenza incidence risk with covariates were $\log ({\theta }_{ij})={\rm{\alpha }}+{\sum }_{k=1}^{n}{\beta }_{k}{x}_{k}+{\nu }_{i}+{\upsilon }_{i}+{\gamma }_{1j}+{\delta }_{ij}$, which included all variables in the univariate analysis models; an intercept (α); a spatially unstructured random effect term (ν_i_); a spatially structured conditional autoregression term (υ_i_); a first-order random walk-correlated time variable (γ_1j_); and an interaction term for time and place (δ_ij_).

**Figure 6:**
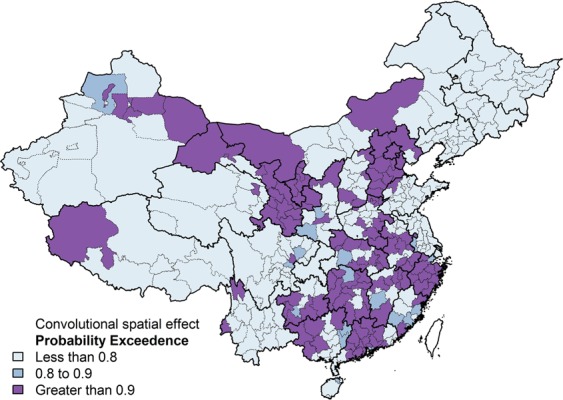
Map of the posterior probabilities of convolutional spatial relative risk ($${e}^{{\upsilon }_{i}+{\nu }_{i}}$$) > 1.0, spatiotemporal model of influenza incidence risk with covariates, China Prefectures, 2005–2018. Note: The linear terms in the model of spatiotemporal model of influenza incidence risk with covariates were $$\log ({\theta }_{ij})={\rm{\alpha }}+{\sum }_{k=1}^{n}{\beta }_{k}{x}_{k}+$$$${\nu }_{i}+{\upsilon }_{i}+{\gamma }_{1j}+{\delta }_{ij}$$, which included all variables in the univariate analysis models; an intercept (α); a spatially unstructured random effect term (ν_i_); a spatially structured conditional autoregression term (υ_i_); a first-order random walk-correlated time variable (γ_1j_); and an interaction term for time and place (δ_ij_).

The convolutional spatial risk term, which includes both the spatially structured conditional autoregression term (υ_i_) and the spatially unstructured random effect term (ν_i_) at the prefecture level, identified areas at increased risk of influenza throughout the 14-year study period (Fig. 5). Posterior probabilities for an area’s spatial risk estimate exceeding 1.0 are presented in Fig. 6.

The proportion of the total spatial heterogeneity explained by the spatially structured conditional autoregression term was 73.51%.

After adjusting for fixed covariate effects and time random effects, both the map of the spatial structured term and the convolutional spatial term show that high-risk areas clustered in the central part of China and the lowest-risk areas in the east, northwest and southwest. The higher-risk prefectures were mostly distributed in Guangdong, Guangxi, Guizhou, Hunan, Jiangxi, Zhejiang, Hubei, Anhui, Henan, Hebei, Beijing, Tianjin, Gansu, Ningxia, and Inner Mongolia. The lower-risk areas in the east included some prefectures in the Shandong peninsula and the prefectures of Heilongjiang, Liaoning, and Jilin provinces in the northeast. The northwest areas are composed of prefectures in Tibet, Qinghai and Xinjiang, while the southwest areas include Chongqing and prefectures in Sichuan and Yunnan provinces.

#### **The temporal trend**

**Figure 7:**
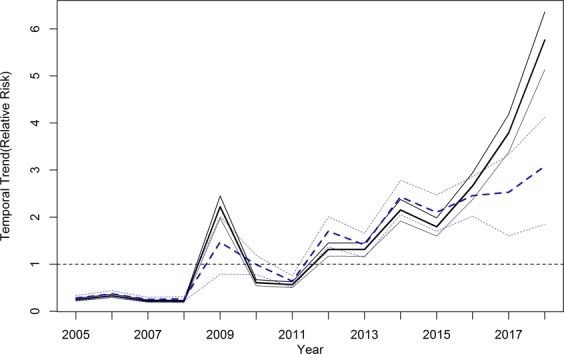
Temporal trend term, spatiotemporal models of influenza risk with and without covariates, China prefectures, 2005–2018. Blue lines: relative risks of years and their 95% confidence intervals in the spatiotemporal model with covariates. Black lines: relative risks of years and their 95% confidence intervals in the spatiotemporal model without covariates.

The relative risks of the 14-year study period, holding the covariates and spatial risk constant, were calculated by exponentiating the marginal first-order random walk-correlated time term (γ_1j_) in the spatiotemporal models of influenza risk with and without covariates. For the spatiotemporal model without covariates, an overall increasing trend was found in the temporal trend term in the 14-year study period. The risk of influenza remained low between 2005 and 2008. A steep increase was observed in 2009. It dropped slightly back to a low level and remained stable in 2010 and 2011. A rapid increase was obvious in the last 3 years (Table 3) (Fig. 7).

**Table 3:** Temporal trend term effects, spatiotemporal models of influenza risk with and without covariates, China prefectures, 2005–2018.

Year	Adjusted OR (95% CI)^*^	Adjusted OR (95% CI)^**^
2005	0.245(0.217~0.272)	0.284(0.227~0.337)
2006	0.328(0.291~0.363)	0.369(0.296~0.437)
2007	0.216(0.191~0.239)	0.250(0.199~0.296)
2008	0.210(0.186~0.233)	0.263(0.209~0.313)
2009	2.221(1.980~2.452)	1.469(0.789~2.013)
2010	0.607(0.540~0.671)	0.993(0.780~1.187)
2011	0.566(0.503~0.626)	0.637(0.509~0.755)
2012	1.316(1.172~1.453)	1.703(1.369~2.009)
2013	1.312(1.168~1.449)	1.410(1.142~1.657)
2014	2.151(1.917~2.375)	2.430(2.054~2.782)
2015	1.795(1.599~1.982)	2.101(1.698~2.473)
2016	2.665(2.375~2.942)	2.457(2.021~2.861)
2017	3.790(3.379~4.183)	2.530(1.602~3.325)
2018	5.763(5.137~6.361)	3.083(1.849~4.123)

^*^Adjusted by convolutional spatial term, space-time interaction term, e.g., $\log ({\theta }_{ij})=\alpha +{\nu }_{i}+{\upsilon }_{i}+{\gamma }_{1j}+{\delta }_{ij}$.

^**^Adjusted by convolutional spatial term, space-time interaction term, and covariates, e.g., $\log ({\theta }_{ij})={\rm{\alpha }}+{\sum }_{k=1}^{n}{\beta }_{k}{x}_{k}+{\nu }_{i}+{\upsilon }_{i}+{\gamma }_{1j}+{\delta }_{ij}$.

For the temporal trend term in the spatiotemporal model with covariates, the relative risks in the years from 2005 to 2016 were not significantly different from that in the spatiotemporal model with covariates. The relative risks in the model with covariates in 2017 and 2018 were significantly lower than those in the model without covariates. The lower boundary of the 95% confidence intervals in the model with covariates showed some levelling off in recent years. The differences between the spatiotemporal model with and without covariates indicated that the recent increases in influenza incidence risks could be partially explained by the fixed covariate effects.

#### **Space-time interactions**

**Figure 8:**
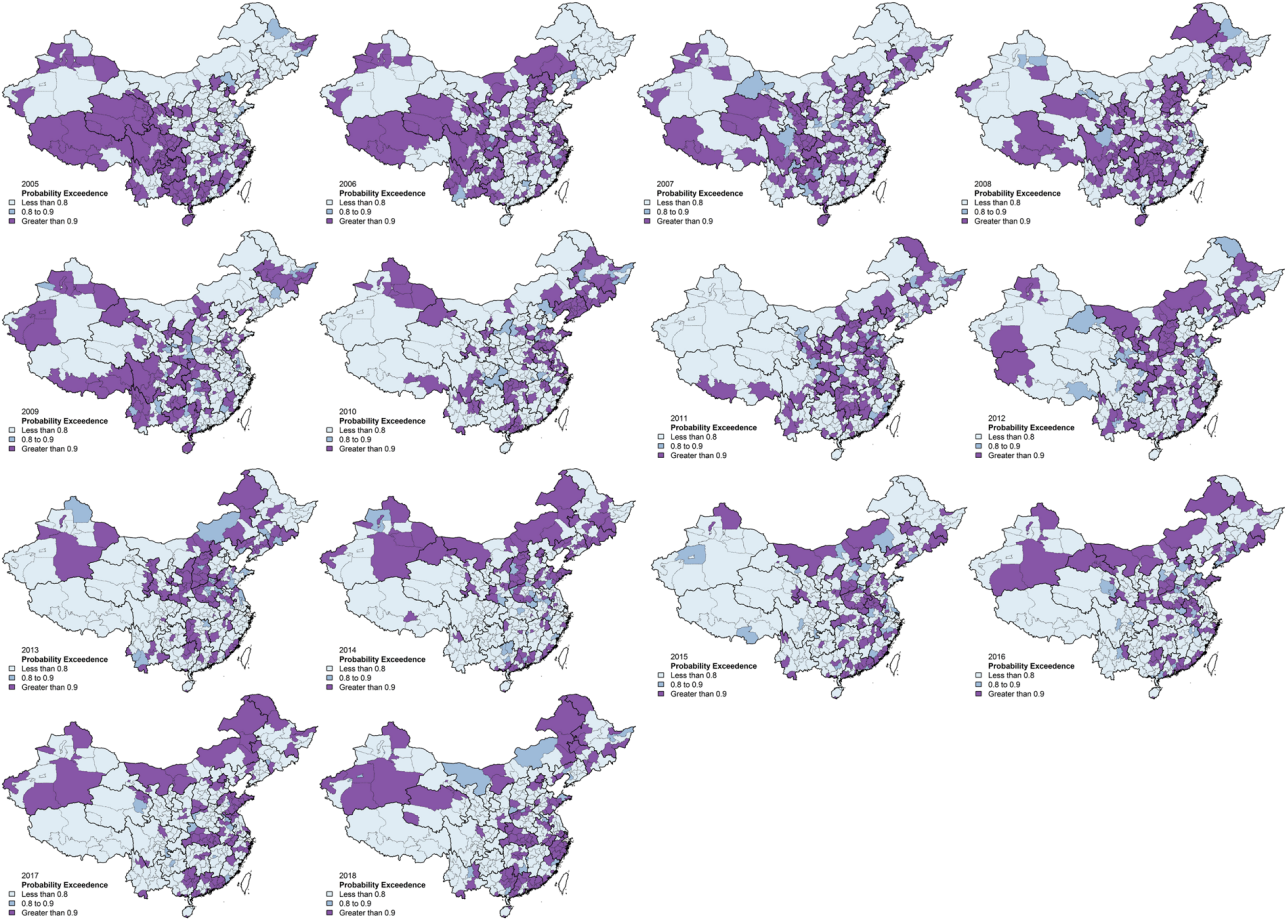
Map of the posterior probabilities of relative risks of space-time interaction terms (${e}^{{\delta }_{ij}}$) > 1.0, spatiotemporal model of influenza incidence risk with covariates, China Prefectures, 2005–2018. Note: The linear terms in the model of spatiotemporal model of influenza incidence risk with covariates were $\log ({\theta }_{ij})={\rm{\alpha }}+{\sum }_{k=1}^{n}{\beta }_{k}{x}_{k}+{\nu }_{i}+{\upsilon }_{i}+{\gamma }_{1j}+{\delta }_{ij}$, which included all variables in the univariate analysis models; which included all variables in the univariate analysis models; an intercept (α); a spatially unstructured random effect term (ν_i_); a spatially structured conditional autoregression term (${{\rm{\upsilon }}}_{{\rm{i}}}$); a first-order random walk-correlated time variable (γ_1j_); and an interaction term for time and place (δ_ij_).

The probability exceedances for the yearly space-time interactions are presented for the study period (Fig. 8). These identify areas with residual spatial risk greater than 1.0 compared to the prefecture-wide risk after the fixed effects, unstructured, spatially structured, and time random effects are held constant. Changing patterns and large variations among the yearly specific spatial distributions are shown in Fig. 8. It is interesting that most of the higher-risk areas were western areas of China before 2009, and most of the higher-risk areas are eastern or northern areas of China after 2009.

## Discussion

Based on the incidence data of influenza gained from the Chinese Notifiable Infectious Disease Reporting System, we used the Bayesian spatiotemporal model in this study to assess the space-time patterns of the influenza epidemic at the prefecture level in mainland China from 2005 to 2018 and explored several factors that may be associated with the changing spatial and temporal patterns in the influenza incidence risk.

### The time trend

An increasing trend of influenza incidence was observed from 2005 to 2018, with a steady level in the first 4 years and a much faster increase in the last 3 years. A sharp increase in 2009 was observed due to the transmission and widespread effect of influenza A (H1N1)pdm09 in China^16,35,36^.

Several potential factors may be associated with the rapid increasing trend of influenza in China.

First, insufficient flu vaccine supplements and a low uptake rate might be associated with an increase in influenza incidence. The results of the final spatiotemporal model showed that every million increase in the number of influenza vaccines approved for sale by the China Food and Drug Administration was associated with a 12.7% decrease in the influenza incidence risk (95% CI = 0.825–0.923). The rapidly increased crude rates of influenza from 2016 to 2018 coincided with a large reduction in the numbers of vaccines approved for sale at the same time. The reductions in the numbers of vaccine supplements were mostly due to the outcomes of vaccine scandals related to improper vaccine storage and production in 2016 and 2018, respectively^9,10,37^.

Previous studies reported that uptake figures of the influenza vaccine averaged 1.9% nationally and 4.3% among urban elderly aged 60 years and above in 9 cities of China during the 2008–2009 and 2011–2012 influenza seasons, respectively^7,8,20^. It is expected that the uptake may be even lower, as people lost their faith in the safety of domestically produced vaccines after the vaccine scandals in China^38^. Our results are consistent with the study in Italy, which reported an association between vaccination coverage decline and influenza incidence among Italian elderly^39^.

Second, currently circulating influenza strains in humans include influenza A (H1N1)pdm09, influenza A (H3N2) and influenza B viruses, (B/Victoria and B/Yamagata)^5,40,41^. Influenza A (H1N1)pdm09 has been reported to be the predominant subtype in recent years according to ILI surveillance and is more likely to be the major cause of regional and widespread outbreaks^40^. Our study showed that for every 10% increase in the rate of influenza A (H1N1)pdm09 among all processed specimens from ILI patients, there was a 19.5% increase in the influenza incidence risk (95% CI = 1.005–1.413). Shu *et al*. reported that the predominant subtype of seasonal influenza A (H1N1) and B/Yamagata could circulate from the south to the north of China from 2006 to 2009^34^. Our study also found that every one degree increase in latitude and longitude was associated with a 1.5% (95% CI = 0.980~0.991) and 0.2% (95% CI = 0.997~0.999) increase in the influenza incidence risk, respectively. This result was consistent with the role of climatic factors in influenza transmission dynamics^20,42^.

Third, the greater effort in influenza surveillance and the use of new technologies may account for the rise in influenza incidence. In recent years, especially after the 2009 pandemic season, influenza surveillance has been expanded worldwide, as recommended by the World Health Organization (WHO)^43–45^. In China, influenza surveillance protocols and technical guidance have been updated in line with new emerging virus subtypes and new detection methods. The major revision in 2009 and 2010 was to include influenza A (H1N1)pdm09 virus infection in the case definition, and updating in 2017 focused on the use of fast detection methods. Our study showed that the new update of the influenza surveillance protocol in 2017 was related to a 65.6% increase in the influenza incidence risk (95% CI = 1.097–2.496) compared to the protocol used in 2005 to 2008. Moreover, the number of sentinel hospitals for ILI reporting increased from 193 in 2005 to 554 in all provinces in 2009^33,34,41^. As CNIDRS includes all sentinel hospitals, sentinel hospitals are likely to report more cases of influenza to CNIDRS. In addition, more hospitals have used electronic health information systems, which may improve both the quantity and quality of data collection and exchange from hospitals to CNIDRS^46–49^.

Fourth, the reporting on influenza A (H1N1)pdm09, avian influenza A (H7N9), highly pathogenic avian influenza (HPAI) H5N1 and avian H6 influenza has increased in recent years^12,18,19,30,50,51^. Constant reports in the media and public health campaigns against the new emerging virus have caused both the government and the public to be more concerned about influenza. The improved public perception of influenza may change people’s health-seeking behaviours, especially in the epidemic seasons^52,53^. Furthermore, enlarged coverage of health care insurance in both urban and rural areas in recent years in China may also induce people to use more health services^54,55^.

A rapid increase in the numbers of airlines and high-speed railway transports in China has been reported in recent years^56^. These factors would make it easy to transmit the influenza virus at a larger scale and in a shorter time across the country^56–58^.

### The spatial pattern

The BYM model includes both a spatial conditional autoregression component and a heterogeneous random effect component. This structure allows us to know how much of the residual disease risk is due to spatially structured variation and how much is unstructured overdispersion^22^.

The spatially structured conditional autoregression term demonstrated areas of spatial patterning and similarity among prefectures. The results of spatially structured variation show a distinguished spatial pattern of risk of influenza across prefectures in China. The highest-risk areas clustered in the middle part of China, while the lowest-risk areas were distributed in the east, northwest and southwest. Different patterns of influenza between the north and south in China were well reported^3,16,20,34,41,59^. In China, the line following the Qinling Mountain range in the west and the Huaihe River in the east is often used to split the mainland into the north and the south^34^. In this study, we observed clustering in both the north and the south in the middle part of China. The unique structured spatial patterns may be attributed to the shared risk factors among the neighbouring areas. This may be associated with similarities in the climatic zone, the predominant subtype of the virus at the time of epidemics, socioeconomic background or lifestyles. The last important factor should not be ignored. Some studies reported that clustering of diseases may be a consequence of spatial heterogeneity in surveillance efforts^60,61^.

### The space-time interaction

The space-time interaction is a random effect term, which is interpreted as the residual effect after the unstructured, spatially structured and time effects are modelled and represent sporadic short-term outbreaks or clusters.

The changes and circulations of virus subtypes may determine the characteristics of the space-time interaction terms. The year 2009 was the critical point according to the results of the spatiotemporal analysis. There are four types of ILI activities: sporadic, local outbreak, regional outbreak and widespread outbreak in FluNet (www.who.int/flunet), Global Influenza Surveillance and Response System (GISRS)^62,63^. Since the first case of influenza A (H1N1)pdm09 was reported on May 9, 2009, in mainland China, the type A (H1N1)pdm09 virus has been detected in all ILI activities according to the data from FluNet.

The yearly ILI activities may be partially associated with the changes and similarities in the patterns of the space-time interactions from 2005 to 2018. From the FluNet data mentioned above, we found that sporadic ILI activities were dominant in 2005, 2006, 2007 and 2008. Correspondently, we found more areas with high relative risk in these 4 years in the space-time term. This implies that the more sporadic the activities are, the larger the variations in the spatiotemporal distribution of the risk of influenza. In contrast, the large outbreaks account for most ILI activities in the years 2009, 2010, 2017 and 2018. Few prefectures were observed to have a relative risk greater than 2 or 3 during that period. Large outbreaks, especially large regional and widespread outbreaks, may reduce the differences in the incidence risk of influenza among the areas and times on a large scale.

### Strengths

This work adds to the existing research on influenza epidemiology in the following ways. First, the study initially presents the spatiotemporal distributions with higher-resolution spatial data than has been reported in China for the last 14 years, which allows more opportunity for focused investigations and interventions. Next, we used the exceedance probabilities instead of the observed risk estimates to identify those areas for which the increased risk was highly unlikely to be due to chance. Then, this study also provided a baseline model that can be extended to include social, economic, ecological, and environmental factors, as well as intervention measures to explore their associations with influenza. Finally, the methods in this study offer practical tools for spatial analysis of other notifiable infectious diseases in CNIDRS.

### Limitations

There are some limitations to this study. CNIDRS is a passive surveillance system, and accessibility to health facilities and patient visit behaviour may affect the number of cases reported. We collected both clinically diagnosed and laboratory-confirmed cases in CNIDRS, so misdiagnosis and misreporting are unavoidable because it is difficult to distinguish influenza from other respiratory viruses without laboratory testing, especially in the non-epidemic seasons.

## Conclusions

This paper outlined the application of the Bayesian spatiotemporal model to assess the relative disease risk of influenza at the prefecture level in mainland China. We observed an increased incidence trend of influenza from 2005 to 2018 that was fairly steady in the first 4 years and increased rapidly in the last 3 years. Clusters of prefectures with high relative risk values concerning influenza incidence were identified in the central part of China. The identification of high-risk areas is especially a priority in China because the limited resources available for disease control need to be focused on the places most in need. We hypothesize that the insufficient flu vaccine supplements, low vaccine uptake, the newly emerging influenza A (H1N1)pdm09 and expansion of influenza surveillance efforts might be the major causes of the dramatic changes in outbreak and spatiotemporal epidemic patterns. Future research with more risk factors at the national and local levels is necessary to explain the changing spatiotemporal patterns of influenza in China.

## Method

### Data sources

Influenza data were obtained from the Chinese Notifiable Infectious Disease Reporting System (CNIDRS). The CNIDRS started in the early 1950s. A web-based online reporting system was developed and put to use in 2004 after the SARS outbreak in 2003^32,64^. To date, 39 notifiable infectious diseases have been included in the reporting system. Hospital doctors are responsible for collecting and reporting the individual case information through the web-based CNIDRS. All 39 notifiable infectious diseases were classified into three categories. A disease in category A should be reported within 2 hours once diagnosed. The diseases in categories B and C should be reported within 24 hours^32,33,64^.

The data on influenza were extracted from the Year-Areas Statistic Tables from 2005 to 2018 in CNIDRS. Four municipalities directly under the central government (i.e., Beijing, Shanghai, Tianjin, and Chongqing without prefecture administrative level) and 344 prefectures were included in the final spatiotemporal analysis.

### Case definitions

Case definitions for influenza and diagnostic criteria are outlined by the National Health and Family Planning Commission of the People’s Republic of China. Both clinically diagnosed cases and laboratory-confirmed cases should be reported to CNIDRS. The influenza A (H1N1)pdm09 cases were added to the general influenza cases in May 2009. Before October 12, 2009, influenza A (H1N1)pdm09 cases only refers to suspected and laboratory-confirmed cases defined in the Guideline for Influenza A (H1N1)pdm09 Treatment (Second Version, 2009). Four types of cases were defined in the third version, 2009. The reported cases include suspected, clinically diagnosed, laboratory-confirmed, and severe cases. The protocols for influenza surveillance and technical guidance were updated in 2009, 2010 and 2017.

### Covariates

In order to account for the changing spatiotemporal patterns of influenza in China, we defined or collected covariates that may be associated with the risk of influenza at the national level. The covariates included the yearly total number of flu vaccines approved for sale by China’s National Institute for Food and Drug Control (NIFDC), the positive rate of influenza A (H1N1)pdm09 among the number of ILI specimens processed, the percentage of influenza A (H1N1)pdm09 among all the positive influenza specimens, and protocol changes. Data on the total number of influenza vaccines released by the China Food and Drug Administration were collected from the National Institute for Food and Drug Control (NIFDC) of China [https://www.nifdc.org.cn/nifdc/fwzn/ppjpqf/index.html]. The numbers of lot releases of influenza vaccines were rescaled to one million doses as one unit. The number of specimens of ILI and the number of influenza A viruses detected by subtype were downloaded from FluNet (www.who.int/flunet), Global Influenza Surveillance and Response System (GISRS). The positive rate of influenza A (H1N1)pdm09 and the percentage of influenza A (H1N1)pdm09 were all rescaled to 10% changes as one unit. The protocols were divided into three stages: 0 for 2005 to 2008, 1 for 2009 to 2016, and 2 for 2017 to 2018. Univariate and multivariate Poisson models were used to calculate crude and adjusted odds ratios (ORs) with 95% confidence intervals (CIs) for covariates, which were further adjusted in the spatiotemporal models hereafter.

### Model specifications for spatiotemporal analysis

The Besag York Mollié (BYM) convolution model was used as a baseline model^22^. Using the notation of Banerjee *et al*.^65^, the BYM model is as follows:

$${y}_{i}\sim Poisson({E}_{i}\ast {\theta }_{i})$$

$$log({\theta }_{i})=\alpha +{\nu }_{i}+{\upsilon }_{i}$$

for *I* ∈ 1: *N*, where

- *N* is the number of areas. The *y*_*i*_ counts of influenza cases in area *i* are independently identically Poisson distributed. *θ*_*i*_ is the risk for area *i*. *E*_*i*_ is the number of expected cases of influenza in area *i*, which acts as an offset.
- *α* quantifies the average incidence risk of influenza in all the prefectures.
- *ν*_*i*_ is a spatially unstructured random effects component that is i.i.d normally distributed with mean zero.
- *υ*_*i*_ is a spatially structured component using an intrinsic conditional autoregressive structure (iCAR).

The random effect for each area *ζ*_*i*_ is thus the sum of a spatially structured component *υ*_*i*_ and an unstructured component *ν*_*i*_. It is termed a convolution prior^22,66^.

The BYM model was extended to include a linear term for space-time interaction and a nonparametric spatiotemporal time trend. Possible random effects specifications for the temporal term include a linear time trend (*β*_*j*_), a random time effect (*γ*_*j*_), a first-order random walk (*γ*_1*j*_), a second-order autoregression (*γ*_2*j*_), etc.^25^.

Four types of interactions are proposed in Knorr-Held (2000)^28^, see Knorr-Held (2000)^28^ for a detailed description. In this study, we assume no spatial and temporal structure on the interaction, and therefore, *δ*_*ij*_ ∼ *Normal*(0; *τ*_*δ*_).

Four candidate models were tested and compared:

1. Model 1, convolution + uncorrelated time (time IID), e.g.,
  $$\log ({\theta }_{ij})=\alpha +{\nu }_{i}+{\upsilon }_{i}+{\gamma }_{j}$$
2. Model 2, convolution + 1st order random walk correlated time (time RW1), e.g.,
  $$\log ({\theta }_{ij})=\alpha +{\nu }_{i}+{\upsilon }_{i}+{\gamma }_{1j}$$
3. Model 3, convolution + 1st order random walk correlated time (time RW1) + uncorrelated time (time IID), e.g.,
  $$\log ({\theta }_{ij})=\alpha +{\nu }_{i}+{\upsilon }_{i}+{\gamma }_{1j}+{\gamma }_{j}$$
4. Model 4, convolution + 1st order random walk correlated time (time RW1) + space-time interaction term with uncorrelated prior for the interaction term

$$\log ({\theta }_{ij})=\alpha +{\nu }_{i}+{\upsilon }_{i}+{\gamma }_{1j}+{\delta }_{ij}$$

In Model 4, the space-time interaction is a random effect term and is interpreted as the residual effect after the unstructured, spatially structured and time effects are modelled and represent sporadic short-term outbreaks or clusters.

Model selection was based on deviance information criteria (DIC), which take into consideration the posterior mean deviance, a Bayesian measure of model fit, and the complexity of the model. A smaller DIC indicates a better fit of the model^67^.

The final linear model consisted of an intercept (α); a vector of national-level explanatory variables $({\sum }_{{\rm{k}}=1}^{{\rm{n}}}{{\rm{\beta }}}_{{\rm{k}}}{{\rm{x}}}_{{\rm{k}}})$ for the yearly total number of lot release of influenza vaccines by the China Food and Drug Administration, the positive rate of influenza A (H1N1)pdm09 among the number of ILI specimens processed, the percentage of influenza A (H1N1)pdm09 among all the positive influenza specimens, and protocol changes; a spatially unstructured random effect term (ν_i_); a spatially structured conditional autoregression term (υ_i_); a first-order random walk-correlated time variable (γ_1j_); and an interaction term for time and place (δ_ij_).

$$\log ({\theta }_{ij})={\rm{\alpha }}+\mathop{\sum }\limits_{k=1}^{n}{\beta }_{k}{x}_{k}+{\nu }_{i}+{\upsilon }_{i}+{\gamma }_{1j}+{\delta }_{ij}$$

The prefecture-specific structured and unstructured spatial risks of influenza compared to the whole spatial risk of all prefectures are obtained by applying an exponential transformation to the components of *ν*_*i*_ and *υ*_*i*_, respectively. The relative risk of space-time interaction is computed by the exponentiation of the term *δ*_i*j*_.

The exceedance probabilities of spatial risk and risk of space-time interaction were also calculated. The exceedance probability represents the posterior probabilities for an area’s spatial risk estimate exceeding some pre-set value and has been proposed as a Bayesian approach to hotspot identification^68,69^.

All spatial models were computed using integrated nested Laplace approximations (INLA), which have been developed as a computationally efficient alternative to MCMC^70^. All spatial analyses were conducted within Microsoft R Open version 3.5 using the R-INLA package (version 18.07.12).

### Ethics approval

The authors assert that all of the procedures contributing to this work comply with the ethical standards of the relevant national and institutional committees on human experimentation and the Helsinki Declaration of 1975 as revised in 2008. This article does not contain any studies of human or animal subjects performed by any of the authors. Since this analysis was based on anonymous aggregated statistical data, patient informed consent and ethical committee approval were not required in China.

### Disclaimer

The views expressed are those of the authors and do not necessarily represent the official policy of the Chinese Center for Disease Control and Prevention.
